# The Ebola Virus VP35 Protein Is a Suppressor of RNA Silencing

**DOI:** 10.1371/journal.ppat.0030086

**Published:** 2007-06-22

**Authors:** Joost Haasnoot, Walter de Vries, Ernst-Jan Geutjes, Marcel Prins, Peter de Haan, Ben Berkhout

**Affiliations:** 1 Laboratory of Experimental Virology, Department of Medical Microbiology, Center of Infection and Immunity Amsterdam, Academic Medical Center of the University of Amsterdam, Amsterdam, The Netherlands; 2 Laboratory of Virology, Wageningen University, Wageningen, The Netherlands; 3 Phytovation B. V., Leiden, The Netherlands; University of Wisconsin-Madison, United States of America

## Abstract

RNA silencing or interference (RNAi) is a gene regulation mechanism in eukaryotes that controls cell differentiation and developmental processes via expression of microRNAs. RNAi also serves as an innate antiviral defence response in plants, nematodes, and insects. This antiviral response is triggered by virus-specific double-stranded RNA molecules (dsRNAs) that are produced during infection. To overcome antiviral RNAi responses, many plant and insect viruses encode RNA silencing suppressors (RSSs) that enable them to replicate at higher titers. Recently, several human viruses were shown to encode RSSs, suggesting that RNAi also serves as an innate defence response in mammals. Here, we demonstrate that the Ebola virus VP35 protein is a suppressor of RNAi in mammalian cells and that its RSS activity is functionally equivalent to that of the HIV-1 Tat protein. We show that VP35 can replace HIV-1 Tat and thereby support the replication of a Tat-minus HIV-1 variant. The VP35 dsRNA-binding domain is required for this RSS activity. Vaccinia virus E3L protein and influenza A virus NS1 protein are also capable of replacing the HIV-1 Tat RSS function. These findings support the hypothesis that RNAi is part of the innate antiviral response in mammalian cells. Moreover, the results indicate that RSSs play a critical role in mammalian virus replication.

## Author Summary

Cells have evolved mechanisms to protect themselves from virus infection. A well-known antiviral mechanism in mammals is the interferon (IFN) response of the innate immune system. In plants, insects, and worms, RNA silencing or RNA interference (RNAi) is a strong antiviral defence mechanism. It is still debated whether RNAi is also used as an antiviral mechanism in mammals. Many mammalian viruses encode essential factors that suppress the innate antiviral responses of the host. Such innate immunity suppressor proteins, or IFN antagonists, have recently been reported to also suppress RNAi in mammalian cells. We now demonstrate that the Ebola virus VP35 protein, a known IFN antagonist, suppresses RNAi in human cells. In addition, VP35 restores the production of an HIV-1 variant with a defective RNAi suppressor Tat protein. These results indicate that RNAi is part of the innate antiviral defence response in mammals and that viruses need to counteract this response in order to replicate. Whereas RNAi and INF act in concert to prevent the infection of mammalian cells, the invading viruses encode a protein that counteracts both defence mechanisms.

## Introduction

An important criterion for productive virus infection is that the virus evades host antiviral immune responses. In plants, insects, and nematodes, the basis of these protective immune responses is formed by the RNA interference (RNAi) mechanism [1–4]. During virus infection, RNAi against the virus is activated by the production of virus-specific double-stranded RNAs (dsRNAs). These virus-specific dsRNAs are processed into small interfering RNAs (siRNAs; a 21-nucleotide dsRNA duplex) by the RNAse III–like endonuclease-denoted Dicer. Subsequently, one strand of the siRNA duplex, the guide-strand, is incorporated into the RNA-induced silencing complex (RISC) to target viral mRNAs bearing complementary sequences for destruction. To overcome this antiviral RNAi response, viruses encode RNA silencing suppressors (RSSs) [5]. For plant viruses, RSSs were first described as pathogenicity factors that contribute to high virus accumulation and disease. One of the best-characterized suppressors is the tombusvirus-encoded P19 protein. This protein, which suppresses RNAi both in plants and mammalian cells, blocks RNAi by binding siRNAs via its dsRNA-binding domain, thereby sequestering the siRNAs from the RNAi pathway [6]. Another way to block RNAi is via inhibition of Dicer activity. For example, the turnip crinkle virus P83 protein was recently shown to specifically block the activity of the Dicer-like 4 protein [7].

Activation of RNAi in mammalian cells, either by transfection of synthetic siRNAs or by endogenous expression of short hairpin RNAs (shRNAs), is a potent new antiviral tool [8]. These findings support the idea that RNAi is part of the innate immune system in mammals. However, in most cases, virus-specific siRNAs could not be detected in virus-infected mammalian cells [9]. So far, virus-specific siRNAs have only been identified in human cells for human immunodeficiency virus type 1 (HIV-1) and the LINE-1 retrotransposon [10–12]. It has been argued that mammalian cells do not need RNAi-based antiviral responses because they have acquired the interferon (IFN) response [13]. However, all other eukaryotes also evolved innate antiviral defence responses. For instance, plants have pattern recognition receptors, and virus recognition leads to apoptosis and the systemic acquired resistance response that is analogous to the IFN response in mammalian cells [14]. Similar to RNAi, the IFN pathway is triggered by cytoplasmic viral dsRNAs and acts as a sensitive and potent antiviral response that is involved in innate and subsequent adaptive immunity.

If RNAi has an antiviral function in mammals, then the infecting viruses should encode RSSs as they do in plant and insect viruses. Recently, several mammalian viruses have been shown to encode viral factors that exhibit RSS activity in animal cells. These factors include the influenza A virus NS1 (NS1), vaccinia virus E3L (E3L), hepatitis C virus Core, primate foamy virus type 1 (PFV-1) Tas, and the HIV-1 Tat proteins, as well as the adenovirus virus-associated RNAs I and II (VAI and VAII) [11,15–18]. Like plant virus RSSs, these suppressors block induced RNAi against reporter gene constructs. Moreover, NS1 and E3L were able to replace the RNAi suppression function of the b2 protein encoded by Flock house virus and to support virus replication in insect cells [17]. Although most of these viral proteins/RNAs are essential for virus replication, their mode of action is largely unknown. We and others have shown that the adenovirus virus-associated RNAs inhibit RNAi by acting as decoy substrates for Exportin 5, Dicer, and RISC [16,19]. HIV-1 Tat is thought to block Dicer activity, whereas NS1 and E3L may sequester dsRNAs and siRNAs [11,17,20]. Strikingly, all RSS proteins from mammalian viruses possess IFN or protein kinase R (PKR) antagonistic properties, suggesting that RNAi and other innate antiviral responses are interrelated [21–24].

Although there is consensus about the fact that viral factors can indeed block RNAi in mammalian cells, there is an ongoing debate about the significance for viral replication [13]. Because non-viral dsRNA-binding proteins such as the bacterial RNase-III protein can also act as RSSs in plant cells, it is important to determine the contribution of RNAi suppression to virus replication [25]. Here, we show that Tat-mediated RNAi suppression is required for HIV-1 virus production. Using defective Tat-minus HIV-1 mutants, we identified the Ebola virus (EBOV) VP35 protein as a potent RNAi suppressor that is functionally equivalent to the HIV-1 Tat RSS function. Furthermore, we show that the E3L and NS1 proteins can also functionally complement the Tat RSS function and rescue virus production of the HIV-1 Tat-minus mutants. These data support the role of RNAi in innate antiviral responses in mammalian cells and the essential role of RNAi suppression in HIV-1 replication.

## Results

### Testing Viral IFN Antagonistic Proteins for RNAi Suppression Activity

**Figure 1:**
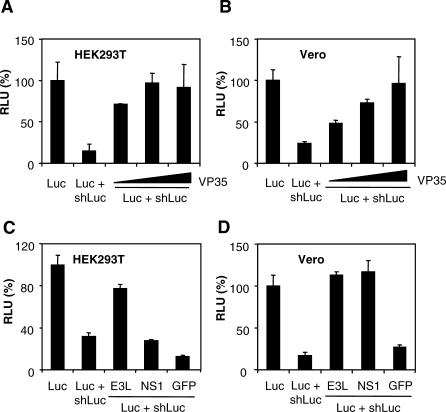
RNAi Suppression by VP35, NS1, and E3L in Mammalian Cells HEK293T cells or Vero cells were transfected with an expression plasmid for firefly luciferase (Luc), a vector expressing an shRNA against luciferase (shLuc), expression constructs for various RSSs (VP35, NS1, E3L), or a control GFP expression plasmid. Luciferase expression was measured at 2–3 d post transfection. (A and B) Effect of VP35 (100, 300, and 600 ng) on the expression of the silenced luciferase reporter in HEK293T cells (A) and (B) Vero cells. (C and D) Effect of NS1, E3L, and GFP (600 ng) on expression of the silenced luciferase reporter in HEK293T cells (C) and in (D) Vero cells.

At present, all RSS proteins identified in mammalian viruses have IFN/PKR antagonistic properties. Previously, it was shown that the EBOV IFN antagonist protein VP35 is capable of restoring the replication of an influenza A virus mutant with a large deletion in the NS1 gene [21,26], raising the possibility that VP35 acts as an RSS in mammalian cells. To investigate the RNAi suppressor activity of EBOV VP35, we assayed whether this protein is capable of suppressing shRNA-mediated silencing of a luciferase reporter. HEK293T cells were co-transfected with expression vectors for luciferase and firefly luciferase–specific shRNA (shLuc) [27], and with an EBOV VP35 expression plasmid containing the human EF1α promoter. Co-transfection of luciferase with shLuc resulted in a strong decrease of luciferase expression (Figure 1A). Addition of the VP35 expression plasmid suppressed the shRNA-mediated RNAi in a dose-dependent manner and restored luciferase expression. To exclude that the observed effect is caused by the IFN antagonistic properties of VP35, its RSS activity was also determined in African green monkey kidney Vero cells that have a defect (IFN^−^) in the IFN pathway [28]. As in HEK293T (IFN^+^) cells, VP35 expression suppressed RNAi in Vero (IFN^−^) cells and rescued luciferase expression (Figure 1B).

Next, we compared the RSS activity of VP35 with that of NS1 and E3L, which were previously demonstrated to have RSS activity in insect and plant cells [17,20,29]. Because insect and plant cells lack the IFN pathway, the RSS activity was determined both in HEK293T (IFN^+^) and Vero (IFN^−^) cells. E3L suppressed RNAi in HEK293T cells, whereas NS1 protein did not (Figure 1C). However, both E3L and NS1 were able to suppress RNAi in Vero cells. The control expression plasmid encoding green fluorescent protein (GFP) did not suppress RNAi-mediated inhibition of luciferase expression (Figure 1D). These data show that VP35, E3L, and NS1 are able to suppress shRNA-induced RNAi in mammalian cells. Possibly, the cell type–dependent RSS activity of NS1 follows from differences in the expression level of certain cellular RNAi co-factors.

### VP35 dsRNA-Binding Capacity Is Required for RSS Activity

**Figure 2:**
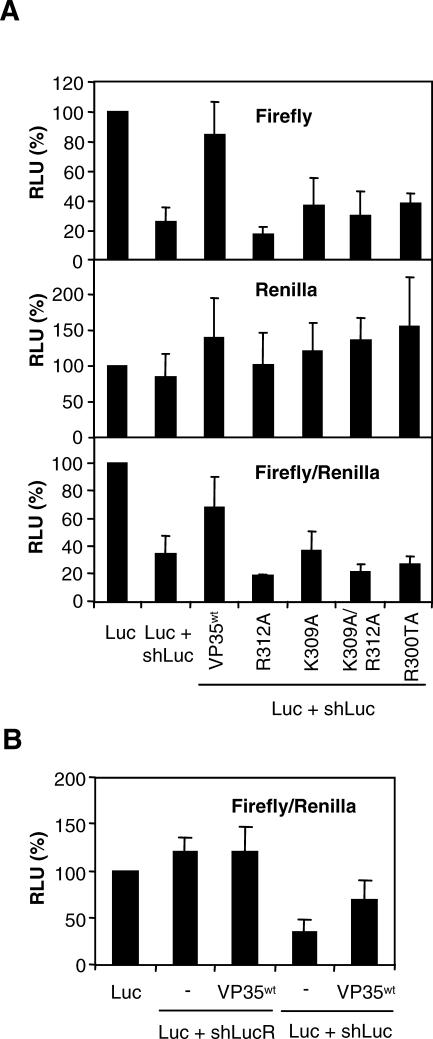
VP35 dsRNA-Binding Capacity Is Required for RSS Activity (A) HEK293T cells were co-transfected with expression vectors for firefly luciferase (Luc), shLuc, the EBOV VP35^wt^, or the various mutants (K309A, R312A, and K309/R12A), or a C-terminal deletion mutant R300T (50 ng). The renilla expression plasmid pRL-CMV was co-transfected as internal control. Luciferase expression was measured at 2–3 d post transfection. From the top down, the panels show the relative expression of firefly luciferase, renilla luciferase, and the ratio firefly/renilla. (B) HEK293T cells were co-transfected with expression vectors for firefly luciferase, renilla luciferase, and either the control shLucR, or the active shLuc in the presence and absence (−) of VP35^wt^ expression plasmid. Luciferase expression was measured at 2–3 d post transfection. Shown is relative luciferase expression corrected for the internal renilla control (firefly/renilla).

VP35 has a dsRNA-binding motif with high similarity to the dsRNA-binding domain of the NS1 protein [30]. Mutational analysis has shown that this VP35 domain is important for suppression of type I IFN responses [30–32]. Because binding of dsRNA is one of the strategies to suppress RNA silencing, we wanted to investigate the importance of this domain in VP35-mediated RSS activity [33,34]. The VP35 dsRNA-binding domain has been identified as ^304^PRACQKSLRPV^314^. We tested two mutants containing a single amino acid substitution, K309A and R312A, and a double mutant, K309A/R312A. Furthermore, we tested the C-terminal deletion mutant R300T, which lacks 40 amino acids. Mutants K309A and K312A have a defect in dsRNA binding, whereas R300T lacks the complete dsRNA-binding domain [30,31]. The wild-type VP35 (VP35^wt^) and the various mutants were cloned in the expression vector pcDNA3.1 containing the cytomegalovirus (CMV) promoter. HEK293T cells were co-transfected with expression vectors for firefly luciferase, shLuc, the wild-type EBOV VP35^wt^, or the various mutants. In addition, a renilla luciferase expression plasmid (pRL-CMV) was co-transfected as internal control. Co-transfection of the firefly luciferase with shLuc resulted in a strong decrease of luciferase expression (Figure 2A). Addition of the VP35^wt^ expression plasmid suppressed shRNA-mediated RNAi and restored luciferase expression. Interestingly, all mutants, in particular R312A, K309A/R312A, and R300T, were unable to rescue luciferase expression (Figure 2A). The internal renilla luciferase control was not affected by the various VP35 expression plasmids, and the firefly/renilla luciferase ratio showed a similar trend as the firefly luciferase data. These results indicate that dsRNA binding is essential for VP35-mediated RNAi suppression in the luciferase assay.

To ensure that the effect of VP35^wt^ is specific for the silenced luciferase, we tested the effect of VP35^wt^ on luciferase expression in the presence of an inactive shRNA against luciferase, shLucR. In shLucR, the hairpin cassette has been inserted in reversed orientation, making it inactive as RNAi inducer [27] (Figure 2B). Co-transfection of the luciferase expression plasmid with shLucR did not affect luciferase expression, whereas shLuc induced a marked reduction in luciferase expression. Co-transfection of the VP35^wt^ expression plasmid did not stimulate luciferase expression in the presence of shLucR, whereas it did enhance luciferase expression in the presence of shLuc. These results show that VP35-stimulated reporter expression is specific for an RNAi-silenced reporter.

### A Second Function of HIV-1 Tat Is Required for Virus Production

**Figure 3:**
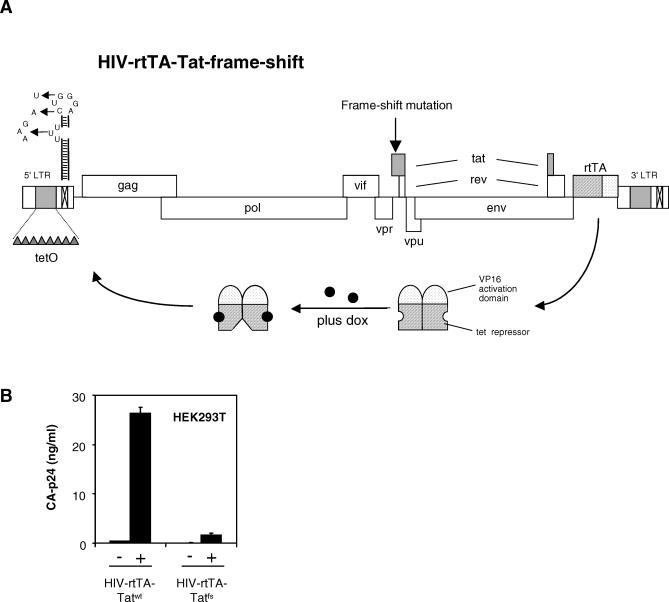
The Tat-Minus HIV-1 Complementation System (A) Schematic of the HIV-rtTA genome. In HIV-rtTA, the Tat-TAR transcriptional axis has been inactivated by mutation of both the Tat protein and the TAR hairpin (as indicated), and replaced by the tetracycline-inducible tetO-rtTA system. For this, the rtTA gene was inserted in place of the Nef gene and eight tetO sites were inserted in the LTR promoter. Upon administration of dox, rtTA can bind to tetO and activate transcription and viral replication. A frame shift mutation was introduced at codon 20 of the Tat gene. (B) Virus production in HEK293T cells transfected with HIV-rtTA-Tat^wt^ or HIV-rtTA-Tat^fs^ in the absence (−) or presence (+) of dox. CA-p24 in the culture supernatant was measured at 3 d post transfection.

RSS proteins derived from plant or insect viruses, but also NS1 and E3L, have been identified by their ability to functionally complement attenuated viruses that lack their own RSS [17,35]. Since it has recently been shown that the HIV-1 Tat protein has RSS activity, we wanted to determine whether EBOV VP35 is capable of functionally replacing the Tat RSS activity in HIV-1. To do this, one needs a Tat-minus virus, but such variants are completely replication impaired due to a transcription defect. However, we previously constructed the HIV-rtTA virus, in which Tat-mediated transcription transactivation is inactivated and replaced by the Tet-On system (Figure 3A) [36,37]. This virus is fully dependent on doxycycline (dox) for gene expression and replication. To test whether the Tat function can be removed in this context, we introduced a frame shift at Tat codon 20 by a single nucleotide deletion in the Tat open reading frame. Virus production was measured in HEK293T cells transfected with the HIV-rtTA molecular clone encoding Tat^wt^ (HIV-rtTA-Tat^wt^) or the frame shift mutant Tat^fs^ (HIV-rtTA-Tat^fs^). Both constructs produced virus in a dox-dependent manner, but Tat^fs^ produced much less virus than HIV-rtTA-Tat^wt^ (Figure 3B). Because HIV-rtTA is not dependent on Tat-mediated transcriptional activation, this result indicates that Tat is needed for an additional function.

### HIV-1 Tat-Mediated Transactivation of Transcription and RNAi Suppression

**Figure 4:**
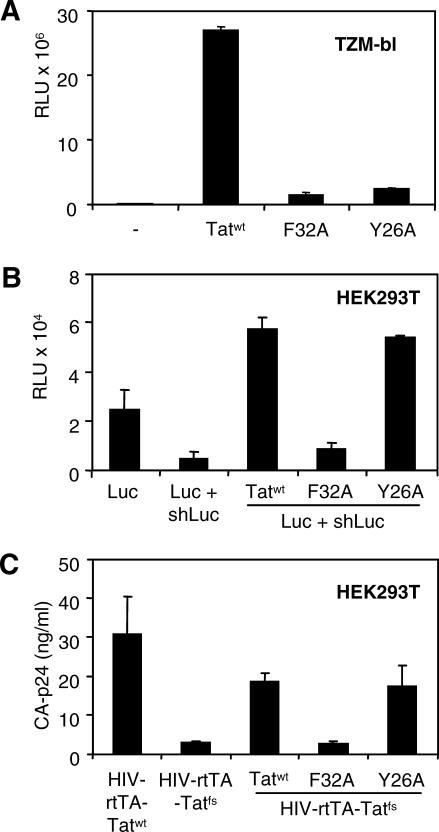
Tat-Mediated RNAi Suppression Is Essential for HIV-rtTA Virus Production (A) TZM-bl cells containing a Tat-responsive firefly luciferase reporter gene under control of the HIV-1-LTR were transfected with pBluescript (−), Tat^wt^, Tat^F32A^, or Tat^Y26A^. Luciferase expression was determined 2 d post transfection. (B) Transfection of HEK293T cells with the luciferase reporter expression plasmid (Luc), the vector expressing an shRNA against luciferase (Luc + shLuc), and expression plasmids for Tat^wt^ , Tat^F32A^, or Tat^Y26A^. Luciferase expression was determined 2 d post transfection. (C) Virus production 2 d post transfection in HEK293T cells transfected with HIV-rtTA-Tat^fs^ and expression plasmids for Tat^wt^, Tat^F32A^, or Tat^Y26A^.

To investigate whether the observed production defect of HIV-rtTA-Tat^fs^ is caused by inactivation of Tat RSS activity, we studied rescue of HIV-rtTA-Tat^fs^ by co-transfection of expression plasmids for Tat^wt^ and two Tat mutants, Tat^F32A^ and Tat^Y26A^ [38]. We first determined the transcriptional activity of Tat^F32A^ and Tat^Y26A^. For this, we used TZM-bl cells that contain the firefly luciferase reporter gene under control of the HIV-1 long terminal repeat (LTR), which is Tat responsive. Only transfection of Tat^wt^ expression plasmid activated luciferase expression, whereas the Tat mutants Tat^F32A^ and Tat^Y26A^ did not stimulate luciferase expression (Figure 4A). We next determined the RSS activity of HIV-1 Tat^wt^, Tat^F32A^, and Tat^Y26A^ using the luciferase RNAi assay. Tat^wt^ and Tat^Y26A^ were capable of suppressing shLuc-mediated silencing of the luciferase expression, but mutant Tat^F32A^ did not suppress RNAi (Figure 4B). Because both mutants express stable proteins that lack transactivation capacity [38], this result indicates that RSS activity can be separated from transcriptional activity, which is in agreement with the results described by Bennasser et al. in 2005 [11].

We next determined whether the production defect of HIV-rtTA-Tat^fs^ in HEK293T cells could be restored by co-expression of Tat^wt^, Tat^F32A^, or Tat^Y26A^. Tat^wt^ and Tat^Y26A^, which are capable of suppressing shRNA-mediated silencing of luciferase expression, rescued the production of HIV-rtTA-Tat^fs^ (Figure 4C). However, Tat^F32A^, which did not suppress RNAi, could not restore virus production. These data indicate that the defect in HIV-rtTA-Tat^fs^ virus production is caused by a *trans*-acting RSS defect, rather than a *cis*-acting defect, of the frame shift mutation.

### Complementation of HIV-rtTA-Tat^fs^ by Heterologous RNAi Suppressor Proteins

**Figure 5:**
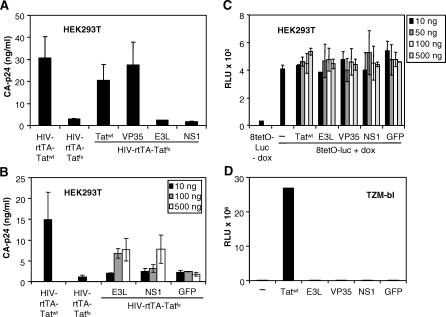
Functional Complementation of the Tat RSS Function by VP35, NS1, and E3L (A and B) HEK293T cells were transfected with HIV-rtTA-Tat^wt^, HIV-rtTA-Tat^fs^, and expression plasmids for (A) Tat^wt^, VP35, E3L, and NS1 (50 ng) or (B) E3L, NS1, and GFP (10, 100, and 500 ng). Virus production was determined 2 d post transfection. (C) To measure transcriptional transactivation capacity of the various proteins, HEK293T cells were transfected with a firefly luciferase reporter under control of the 8tetO promoter (similar to the promoter in the HIV-rtTA constructs), renilla expression vector pRL-CMV, an expression plasmid for rtTA to activate the promoter in the presence of dox, and the indicated amounts of RSSs. Luciferase expression (plotted as firefly/renilla) was determined 2 d post transfection. −, pBluescript (negative control). (D) Transcriptional transactivation capacity of the various RSS proteins was measured using TZM-bl cells that contain a Tat-responsive firefly luciferase reporter gene under control of the HIV-1-LTR. The cells transfected with 0.2 μg of Tat or the indicated RSS expression plasmids. Two to three days after transfection, luciferase expression was measured. −, pBluescript (negative control).

We subsequently tested whether the HIV-rtTA-Tat^fs^ production defect could also be restored by co-transfection of the EF1α expression plasmids for VP35, NS1, or E3L. Indeed, VP35 rescued virus production of HIV-rtTA-Tat^fs^ (Figure 5A). Little RSS activity was measured for E3L and NS1 (Figure 5A), but increasing activity was measured when more RSS DNA was used in the transfection (Figure 5B). In the latter case, a GFP reporter was included as a negative control. To exclude that VP35, E3L, NS1, and Tat^wt^ stimulate HIV-rtTA-Tat^fs^ virus production by inducing promoter activity, we transfected cells with a firefly luciferase reporter under transcriptional control of the 8tetO promoter (the same promoter as in HIV-rtTA-Tat^fs^) with an rtTA expression plasmid and with the different RSS vectors. We measured dox-dependent promoter activity resulting in luciferase expression (Figure 5C). Co-expression of Tat^wt^, E3L, VP35, NS1, or GFP did not further increase luciferase expression, indicating that these proteins do no activate the 8tetO promoter *in trans*. The various RSS proteins also did not activate expression of a luciferase reporter under control of a wild-type HIV-1 LTR promoter in TZM-bl cells (Figure 5D). This shows that the observed rescue of the Tat function by the heterologous RSSs is independent of promoter activation.

**Figure 6:**
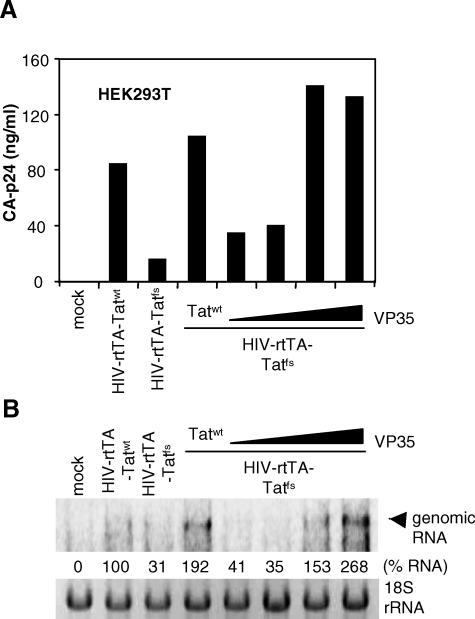
Analyses of HIV-rtTA-Tat^fs^ Genomic RNA Accumulation Complemented with Tat and VP35 (A) HEK293T cells were transfected with HIV-rtTA-Tat^wt^, HIV-rtTA-Tat^fs^, and HIV-rtTA-Tat^fs^ in combination with Tat^wt^ or increasing amounts of VP35. Two to three days after transfection, virus production was measured via CA-p24 ELISA, and total RNA was isolated. (B) Total RNA (10 μg) was run on a denaturing agarose gel, and after blotting, probed with a Nef-specific radiolabeled probe. Relative genomic RNA accumulation was quantified by phosphorimaging and is indicated below. The RNA accumulation of the HIV-rtTA-Tat^wt^ was set at 100%.

To confirm that the observed rescue of virus production corresponds with an increase in viral mRNAs, we analysed viral RNA accumulation by Northern blot analyses. First, rescue of HIV-rtTA-Tat^fs^ virus production by Tat^wt^ and VP35 was determined by measuring CA-p24 production in the supernatant (Figure 6A). The amount of full-length genomic RNA of HIV-rtTA-Tat^fs^ accumulating in transfected cells was reduced when compared to that of HIV-rtTA-Tat^wt^ (Figure 6B). In accordance with the CA-p24 values, co-transfection of Tat and VP35 restored the amount of full-length HIV-1 RNA.

### VP35 dsRNA-Binding Capacity Is Required for *trans* Complementation of HIV-rtTA-Tat^fs^

**Figure 7:**
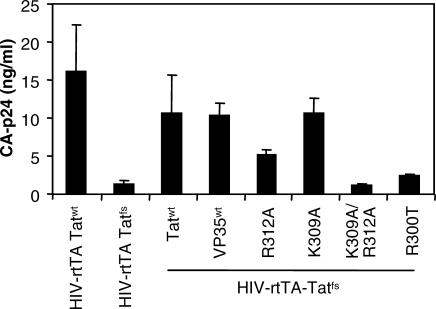
VP35 dsRNA-Binding Capacity Is Required for *trans* Complementation of HIV-rtTA-Tat^fs^ HEK293T cells were transfected with HIV-rtTA-Tat^wt^, HIV-rtTA-Tat^fs^, and expression plasmids for Tat^wt^, VP35^wt^, or the various VP35 mutants (K309A, R312A, and K309/R12A), or a C-terminal deletion mutant R300T (50 ng). Virus production was determined 2 d post transfection.

Next, we wanted to determine whether the VP35 dsRNA-binding capacity is required for the complementation of HIV-rtTA-Tat^fs^. HEK293T cells were transfected with either HIV-rtTA-Tat^wt^ or HIV-rtTA-Tat^fs^, and expression plasmids for Tat^wt^, VP35^wt^, and the VP35 mutants K309A, R312A, K309A/R312A, and R300T (Figure 7). Tat^wt^, VP35^wt^, and K309A were able to rescue HIV-rtTA-Tat^fs^ production, and co-transfection of mutant R312A partially restored HIV-rtTA-Tat^fs^ virus production. In contrast, mutants K309/R312A and R300T were unable to rescue virus production. These results correlate with the ability of the various VP35 mutants to suppress RNAi in the luciferase assay. Thus, the data confirm the importance of RNAi suppression in HIV-1 production and that the dsRNA-binding capacity of VP35 is essential for this activity.

## Discussion

Although the importance of antiviral RNAi responses in plants, nematodes, and insects is firmly established, it is still under debate whether RNAi has a similar function in mammalian cells. If RNAi is a protective antiviral response in mammals, then virus infection should trigger the production of virus-specific siRNAs; inhibition of RNAi should affect virus replication, and viruses should have evolved factors that suppress RNAi [13]. To date, there are only three examples of virus-specific siRNAs accumulating in infected mammalian cells. The human retrotransposon LINE-1 was shown to be inhibited by transposon-specific siRNAs, which is similar to what was previously described for transposon silencing in Caenorhabditis elegans [10,12,39]. Furthermore, there is data suggesting accumulation of virus-specific siRNAs during HIV-1 replication [11]. However, virus-specific siRNA accumulation in mammalian cells appears to be relatively low compared to that in plant and insect cells. The reason for this has remained unclear. One explanation could be the lack of host RNA-dependent RNA polymerase (RdRp) activity in mammalian cells. In plants and insects, RdRp activity is responsible for amplification of the RNAi signal. In combination with viral RSS activity, the absence of RdRp activity could explain the low siRNA levels in mammalian cells in comparison to those in plant, insect, or nematode cells. Another possibility is that antiviral RNAi in mammalian cells is initiated by cellular microRNAs rather than by de novo production of siRNAs [15]. This has been suggested for the primate retrovirus PFV-1, where the cellular miR-32 was found to target PFV-1 sequences. PFV-1 overcomes microRNA-mediated antiviral pressure by the expressing the RSS Tas protein. In principle, viruses could also escape from antiviral microRNA pressure by acquisition of one or a few point mutations within the target sequence [40,41]. It is therefore likely that there is a more general RNAi response against PFV infection that necessitates the presence of an RSS.

Little is known about enhanced virus replication in cells with a defective RNAi mechanism. However, recently it has been reported that HIV-1 replication is increased in human cells in which Dicer and Drosha expression is inhibited [42]. This suggests that RNAi indeed plays a central role in anti-HIV-1 defence responses in human cells. Another report describes enhanced accumulation of the mammalian vesicular stomatitis virus in C. elegans with a defective RNAi machinery, which confirms that RNAi has an antiviral activity in nematodes [4]. The other indication for RNAi-mediated antiviral activity in mammals is the fact that a number of mammalian viruses encode potent RSSs. However, it is still unclear to what extent these suppressors contribute to virus replication, because these factors have multiple functions that are difficult to separate. Moreover, the relevance of RSS activity measured in reporter assays has been questioned because non-specific binding of siRNAs by overexpression by dsRNA-binding proteins might also result in RNAi suppression [13]. Therefore, it is essential to test the importance of RSS activity in a viral context instead of in reporter assays. Using the HIV-rtTA virus as a tool to investigate RNAi suppression, we were able to separate the transactivation function of HIV-1 Tat from its RSS function. We showed that Tat-mediated RSS activity is essential for HIV-1 production without possible side effects of Tat-mediated transcriptional transactivation. These data are in agreement with data published by Bennasser et al. in 2005 [11]. The HIV-rtTA virus was used as a tool to identify EBOV VP35 protein as an RSS that can complement this Tat function. Both HIV-1 Tat and EBOV VP35 were found to complement HIV-rtTA-Tat^fs^ production efficiently at low concentrations, indicating specific RSS activity. Moreover, RSSs encoded by other mammalian viruses are also able to complement the Tat RSS activity. The results presented here support the importance of RNAi in innate antiviral defence responses in mammalian cells and the essential function of RSSs in mammalian virus replication.

In plants, insects, and nematodes, antiviral RNAi responses are triggered by virus-specific dsRNAs. In mammalian cells, virus-specific dsRNAs induce the IFN pathway via members of the Toll-like receptor family, or via a replication-dependent pathway involving the cytoplasmic dsRNA sensors RIG-I/MDA5 (retinoic-acid-inducible protein I/melanoma-differentiation-associated gene 5) [43,44]. Other antiviral proteins that are induced by dsRNA include the 2′-5′ oligoadenylate cyclase (2′-5′ OAS)/RNAseL and PKR [45,46]. Since RNAi, IFN responses, and 2′-5′ OAS/RNAseL/PKR are triggered by dsRNA, it is likely that these pathways cooperate in the innate antiviral defence response. The helicases RIG-I/MDA5 are candidate proteins that could link antiviral RNAi and IFN responses because they can be activated by siRNAs [44,47].

**Figure 8:**
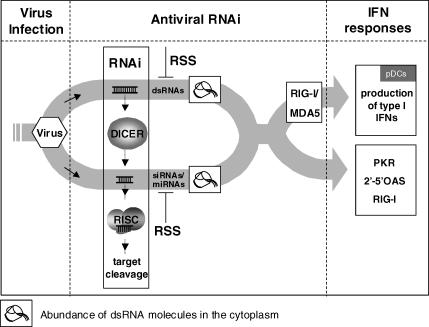
Antiviral RNAi Activity and IFN Responses Function together in Innate Antiviral Defences During virus replication, virus-specific dsRNA molecules are generated that are recognized by Dicer and processed into siRNAs. One strand of the siRNA, the guide-strand, is loaded into RISC, which targets viral RNAs for destruction. To escape this antiviral pressure, viruses encode RSS factors that block the RNAi pathway. When virus-specific dsRNA molecules and siRNAs accumulate above a certain threshold, and consequently can no longer be masked by the viral RSS, the cytoplasmatic dsRNA sensors RIG-I/MDA5, PKR, and 2′5′ OAS/RNAseL are activated. This results in the activation of range antiviral responses, including the production of type I IFNs, general translational inhibition, and RNA degradation. This model clarifies why RSSs like NS1, VP35, and E3L have been identified as IFN antagonists.

In agreement with this model is the recent observation that EBOV VP35 inhibits RIG-I induced activation of type I IFN responses [31]. In part, this inhibition is dependent on dsRNA-binding capacity of VP35 [31]. The K309A mutant was shown to have a partial defect in IFN antagonistic activity, whereas the other mutants, R312A, K309A/R312A, and R300T, were severely impaired in blocking IFN responses. Similarly, our assays show that mutation of the dsRNA-binding domain results in a loss of RNAi suppression activity. Only mutant K309A showed partial RSS activity and was able to complement HIV-rtTA-Tat^fs^ production. These data suggest that VP35-mediated RNAi suppression and VP35-dsRNA-dependent IFN antagonistic properties are linked. We therefore propose that VP35 is able to sequester virus-specific siRNAs or dsRNA precursors of siRNA, resulting in suppression of an antiviral RNAi response that acts upstream of the 2′-5′ OAS/RNAseL/PKR and IFN pathways. In this scenario, the amount of cytoplasmic virus–specific siRNAs needs to reach a certain threshold level before RIG-I/MDA5 and the IFN pathway are activated (Figure 8). In this way, virus-specific siRNAs would function as signal molecules for activation of the IFN response.

Virus-mediated RNAi suppression therefore has two functions. First, it suppresses the RNAi response that acts as the first line antiviral defence. Second, the inhibition of siRNA accumulation prevents RIG-I/MDA5-mediated activation of the IFN pathway. Important in this respect are the immature plasmacytoid dendritic cells (PDCs), which play a predominant role in antiviral immunity. If the accumulation of virus-specific siRNAs in these cells is blocked by viral RSS activity, there will be no trigger to induce the secretion of class I IFNs that in turn induce a range of antiviral genes, resulting in the antiviral state of the neighbouring cells, dendritic cell maturation, and subsequent adaptive immunity [48]. This results in a delayed onset of the immune responses, and neighbouring cells will remain permissive to the invading virus for a prolonged time [49].

Evasion of antiviral immune responses is a key process during virus replication. The results presented here suggest that RNAi plays an important role in innate antiviral defences and that HIV-1 needs to counter this mechanism in order to replicate. The fact that many RSS proteins also have IFN antagonistic properties [50–56] supports the idea that RNAi and IFN responses work together against invading viruses. It remains to be determined how the RNAi and IFN responses cooperate in mechanistic terms. In addition, future experiments should reveal the precise viral signature that activates the antiviral RNAi response in mammals.

Although viral RSS factors will be able to counter RNAi during natural infections, these factors will likely not reduce the effectiveness of RNAi-based antiviral therapeutics. High concentrations of exogenous synthetic siRNAs, for example, to block EBOV replication will easily saturate the VP35 RSS activity, rendering VP35 ineffective, and subsequently still inhibit virus replication [57]. Similarly, plant viruses that encode RSS factors can also be silenced by RNAi, as can viruses such as HIV-1, influenza, hepatitis C virus, and Flock house virus that encode RSS factors.

Besides revealing novel aspects of the virus–host interaction, the *in trans* complementation of viruses lacking their RSS by heterologous RSSs creates opportunities for improving production of virus particles in mammalian cells. Moreover, this phenomenon could be used to develop a new generation of live attenuated viral vaccines that improves upon the current antiviral measures.

## Materials and Methods

### Construction of expression plasmids.

The NS1 (from influenza A virus strain PR8 [20]), VP35 (from Ebola virus strain Zaire), E3L (from vaccinia virus strain Ankara), and EGFP open reading frames were cloned into the mammalian expression vector pEF5-V5-DEST containing human EF1α promoter using GATEWAY technology (Invitrogen, http://www.invitrogen.com). The constructs for VP35^wt^ and the various VP35 mutants contain the CMV promoter (pcDNA3.1-VP35^wt^, K309A, R312A, K309A/R312A, and R300T) and were a kind gift from S. T. Nichol [30]. The HIV-1 Tat expression plasmids, pcDNA3-wtTat/pKV-wtTat, and the mutants, pcDNA3-Y26A and pKV-F32A, were described previously [38]. HIV-rtTA-Tat^fs^ was created by deletion of A58 in the Tat open reading frame of HIV-rtTA-Tat^Y26A^ (variant KYK in [37]), thus creating a frame shift at codon 20.

### Cell culture and transfections.

Human embryonic kidney (HEK293T) cells and African green monkey kidney Vero cells were grown as a monolayer in DMEM (Gibco BRL, http://www.invitrogen.com) supplemented with 10% fetal calf serum (FCS) (Hyclone, http://www.hyclone.com), minimal essential medium with non-essential amino acids, penicillin (100 U/ml), and streptomycin (100 μg/ml) at 37 °C and 5% CO_2_. One day before transfection, cells were trypsinized, resuspended in DMEM, and seeded in 24-well plates at a density of 1.5 × 10^5^ cells per well. At the time of transfection, the cells were 60%–70% confluent. The transfection was performed in duplicates using Lipofectamine 2000 (Invitrogen) according to the instructions of the manufacturer. For the luciferase RNAi assay, cells were transfected with 100 ng of luciferase-expressing plasmid pGL3 (Promega, http://www.promega.com) and 10 ng of expression plasmid shLuc (pShh1-Ff1), from which an shRNA against luciferase is expressed under control of the U6 promoter [27]. Cells were lysed 2–3 d post transfection in 150 μl of 1x Passive Lysis Buffer (Promega) by shaking for 30 min at room temperature. The cell lysate was centrifuged for 5 min at 1,500 rpm, and luciferase expression was measured in 10 μl of supernatant with the luciferase reporter assay system (Promega).

For the complementation studies, 100 ng of HIV-rtTA-Tat^fs^ was transfected with the indicated amounts of RSS plasmid. The total amount of DNA was brought to 1 μg using pBluescript (Stratagene, http://www.stratagene.com). Two to three days after transfection, virus production was determined by measuring CA-p24 levels by enzyme-linked immunosorbent assay (ELISA).

For the testing of the effect of Tat and the various RSS proteins on the 8tetO promoter, we transfected HEK293T cells with 20 ng of 8tetO-luc expression plasmid containing a luciferase reporter under control of the 8tetO promoter (the same promoter as in HIV-rtTA-Tat^fs^), 0.4 ng of rtTA expression plasmid, 0.5 ng of pRL-CMV, and the different RSS vectors. Two to three days after transfection, luciferase expression was measured.

### Transactivation assay.

Transcriptional transactivation capacity of Tat, Tat mutants, and the various RSS proteins was measured using TZM-bl cells. These cells were obtained from the NIH AIDS Research and Reference Reagent Program (also termed JC53-BL cells; catalog number 8129; https://www.aidsreagent.org). TZM-bl cells are genetically modified Hela cells that express CD4, CXCR4, and CCR5 and contain the firefly reporter gene under control of the HIV-1 LTR, which is Tat responsive. The cells were seeded similarly to the HEK293T cells and transfected with 0.2 μg of Tat or RSS expression plasmid, and luciferase expression was measured 2–3 d after transfection.

### Northern blot analyses.

HEK293T cells (T25 flask) were transfected with 6.6 μg of HIV-rtTA-Tat^wt^, HIV-rtTA-Tat^fs^, and HIV-rtTA-Tat^fs^ in combination with 0.132 μg of Tat^wt^ or 0.132/0.66/1.32/6.6 μg of VP35 expression plasmid. Dox was added to the medium to activate HIV-rtTA virus production. Two to three days post transfection, virus production was measured via CA-p24 ELISA, and total RNA was isolated using the mirVana RNA isolation kit (Ambion, http://www.ambion.com). For detection of genomic HIV-1 RNAs, gel electrophoresis of 10 μg of total RNA was performed on a denaturing formaldehyde, 1% agarose gel. RNA was transferred to a positively charged nylon membrane (Boehringer, http://www.boehringer-ingelheim.com) via capillary blotting and crosslinked to the membrane with a UV crosslinker (Stratagene). A 19-nt LNA-modified oligonucleotide complementary to the HIV-1 Nef gene was used as a probe, which was 5′ end labeled using the kinaseMax kit (Ambion) in the presence of 1 μl of [γ-^32^P]ATP (0.37 MBq/μl; Amersham Biosciences, http://www.gelifesciences.com) and purified over a MicroSpin G-25 column (Amersham Biosciences). Prehybridization and hybridization was performed in Ultrahyb buffer (Ambion) at 60 °C for 30 min and 18 h, respectively. The membrane was washed twice for 15 min at 60 °C with high-stringency buffer (0.2x SSC, 0.2% SDS). Images were obtained using the Typhoon Trio phosphor imager (Amersham Biosciences).

## Supporting Information

### Accession Numbers

The GenBank (http://www.ncbi.nlm.nih.gov/Genbank/index.html) accession numbers for the proteins discussed in this article are E3L (from vaccinia virus) (U94848), NS1 (from influenza A virus) (EF467817), and VP35 (from Ebola virus) (AF086833).

## Abbreviations

CMV: cytomegalovirus
dox: doxycycline
dsRNA: double-stranded RNA
EBOV: Ebola virus
E3L: vaccinia virus E3L
GFP: green fluorescent protein
HIV-1: human immunodeficiency virus type 1
IFN: interferon
LTR: long terminal repeat
NS1: influenza A virus NS1
PFV-1: primate foamy virus type 1
PKR: protein kinase R
RIG-I/MDA5: retinoic-acid-inducible protein I/melanoma-differentiation-associated gene 5
RNAi: RNA interference
RSS: RNA silencing suppressor
shRNA: short hairpin RNA
shLuc: firefly luciferase–specific shRNA
siRNA: small interfering RNA
